# Longitudinal test-retest neuroimaging data from healthy young adults in southwest China

**DOI:** 10.1038/sdata.2017.17

**Published:** 2017-02-14

**Authors:** Wei Liu, Dongtao Wei, Qunlin Chen, Wenjing Yang, Jie Meng, Guorong Wu, Taiyong Bi, Qinglin Zhang, Xi-Nian Zuo, Jiang Qiu

**Affiliations:** 1 Key Laboratory of Cognition and Personality (SWU), Ministry of Education, Chongqing 400715, China; 2 Faculty of Psychology, Southwest University, Chongqing 400715, China; 3 Donders Institute for Brain, Cognition, and Behaviour, Radboud University Medical Centre, Nijmegen 6525 EZ, The Netherlands; 4 Key Laboratory of Behavioral Science, Laboratory for Functional Connectome and Development and Magnetic Resonance Imaging Research Center, Institute of Psychology, Chinese Academy of Sciences, Beijing 100101, China; 5 Department of Psychology, School of Education Science, Guangxi Teachers Education University, Nanning 530000, China

**Keywords:** Functional magnetic resonance imaging, Neuroscience, Human behaviour, Brain imaging

## Abstract

Multimodal magnetic resonance imaging (mMRI) has been widely used to map the structure and function of the human brain, as well as its behavioral associations. However, to date, a large sample with a long-term longitudinal design and a narrow age-span has been lacking for the assessment of test-retest reliability and reproducibility of brain-behavior correlations, as well as the development of novel causal insights into these correlational findings. Here we describe the SLIM dataset, which includes brain and behavioral data across a long-term retest-duration within three and a half years, mMRI scans provided a set of structural, diffusion and resting-state functional MRI images, along with rich samples of behavioral assessments addressed—demographic, cognitive and emotional information. Together with the Consortium for Reliability and Reproducibility (CoRR), the SLIM is expected to accelerate the reproducible sciences of the human brain by providing an open resource for brain-behavior discovery sciences with big-data approaches.

## Background & Summary

During the previous two decades, cognitive neuroscientists, psychiatrists, biomedical engineers and neurologists have witnessed the advent and advancement of noninvasive brain techniques, which enable the visualization and analysis of human brain structure and function in detail^1,2^. Among these techniques, Magnetic Resonance Imaging (MRI) has a unique advantage in the investigation of the human brain because the same scanner may perform the data collection of various image modalities, which each provide information on brain structure, function or metabolism. Neuroanatomical images of the human brain, which are also acquired in standard medical exams, may be rapidly collected, at a low cost using MRI. Recent studies have demonstrated that inter-individual variability in human behavior and cognition can be predicted from local brain structures^3^, which highlights the potential use of structural brain images in cognitive and psychological science. Resting-state fMRI (rfMRI) and diffusion weighted imgaing (DWI) may be used to capture spontaneous brain activity and white matter integrity, and more importantly, to describe the functional and anatomical connectivity between brain regions.

Investigators may currently obtain access to high-quality, open-access MRI databsets from every corner of the earth via the Internet, as a result of the trend that research groups tend to upload their MRI data and provide public access. These databases include the Human Connectome Project^4^, the Cambridge Centre for Ageing and Neuroscience (Cam-CAN)^5^, the Pediatric Imaging, Neurocognition, and Genetics (PING) Data Repository^6^, the Consortium for Reliability and Reproducibility (CoRR)^7^, the Autism Brain Imaging Data Exchange (ABIDE)^8^, the Brain Genomics Superstruct Project^9^. These open-access MRI databases act as catalysts for investigating the scientific questions that could not be previously investigated. For example, using data from the Human Connectome Project, researchers determined that patterns of functional connectivity may be used as a ‘connectome fingerprint’ to identify an individual at the participant level^10^, that subjects’ functional connectomes can be linked to 280 behavioral and demographic measures in a single holistic multivariate analysis^11^ and that intrinsic functional connectivity can predict individual differences in task-evoked brain activation^12^. However, these MRI datasets have their limitations. For example, most databases have only released the cross-sectional MRI data to date, and the age span of their subjects is substantial.

Here, we describe the data generated in the Southwest University Longitudinal Imaging Multimodal (SLIM) study, which is available for research through the International Data-sharing Initiative (INDI, http://fcon_1000.projects.nitrc.org/). The Southwest University Longitudinal Imaging Multimodal (SLIM) Dataset comprises a large sample multi-modal (sMRI, rs-fMRI, DWI and behavioral), longitudinal (average interval days=817.87 days) investigation of the neural underpinnings and development of creativity and emotion. The goal of the SLIM was to investigate the neural substrate of individual differences in a variety of psychological traits, mainly the creativity and risk factors of affective disorders, using both the cross-sectional and longitudinal data. Of note, a subgroup of the subjects (time point 1 and time point 2) in this dataset has been shared as a part of the Consortium for Reliability and Reproducibility (CoRR)^7^. However, we did not release the recently collected time point 3 images and just released the demographic variables, but no other behavioral variables in the CoRR. Thus, we released the neuroimaging data (at three time points) and rich samples of behavioral assessments as the SLIM. The whole dataset together could allow people to investigate the scientific questions that can only be explored using the dataset as a whole.

Two features of the SLIM Data Repository make it unique. First, the age span of the participants is small because all subjects in this project are undergraduate students, which reduces the individual differences of the brain structure and function that may arise with the age. Second, there are several cross-sectional open-access imaging data resources worldwide; however, an open-access longitudinal neuroimaging data repository remains rare. The long-term longitudinal data provided by the SLIM can allow investigators to track the brain changes during a long period time using the images from three time points. For example, a recent work from our lab tired to investigate the relationship between functional brain network and trait/state anxiety. We identified functional brain networks whose strength remain relative stable and other brain networks whose strength change dramatically across different scanning sessions. We hypothesized that stable brain networks would associate with the trait anxiety while the dynamic brain networks would relate to the state anxiety. Finally, this study collected a broad range of behavioral data, such as personality, emotion, creativity, and social support, and repeatedly measured variables of interest. This approach enables researchers to investigate the development of these psychological traits or abilities during early adulthood using the neuroimaging data.

In the following sessions, we briefly describe the participant recruitment and data collection, the data record, technical validation and sharing and access policy.

## Methods

### Overall design

**Figure 1:**
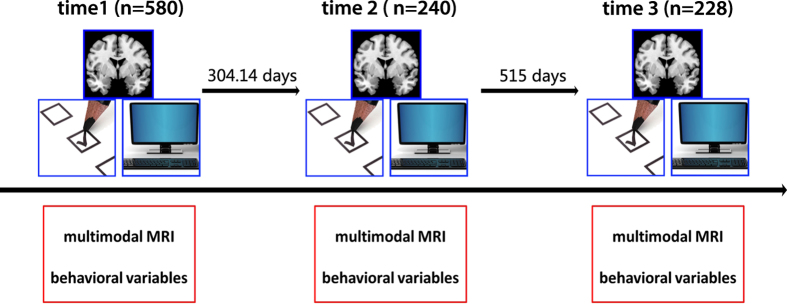
Overall study design. The dataset includes multimodal MRI data and behavioral variables at three time points. The number of subjects at three time points included 580, 240, and 228, respectively.

The data collection was initiated in November 2011 and was terminated in January 2015. The general information is shown in the Fig. 1. The dataset includes data from 121 subjects with all three sessions, 211 subjects with two sessions, and 263 subjects with only one session. At the time point 1, the average age of all the subjects is 20.08 (range 17–27) and the median is 20. There are 323 females, 257 males (time point 1) and 121 females, 119 males (time point 1), and 135 females, 93 males (time point 1) participated in the scanning. The average number of days between the first scan and the third scan are 817.87 days.

### Participant recruitment

The participants were initially recruited through flyers, online advertisements, and face-to-face communications on the campus of Southwest University. Young adults were screened as eligible for the SLIM if they were freshman or sophomores and fluent in Chinese. The exclusion criteria included: (1) MRI related exclusion criteria, which included claustrophobia, metallic implants, Meniere’s Syndrome and a history of fainting within the previous 6 months; (2) current psychiatric disorders and neurological disorders; (3) use of psychiatric drugs within the three months prior to scanning; (4) pregnancy; or (5) a history of head trauma. We invited the subjects who completed the first scan to the follow-ups via the telephone, text or e-mails. Every subject was paid for participation (about 25–30 dollars for each MRI scan and 10 dollars for each 2-hour behavioral test). This study was approved by the Research Ethics Committee of the Brain Imaging Center of Southwest University. Informed written consent was obtained from each subject, and we also obtained informed written consent from the guardians of the two youngest participants (aged 17 years old) who were their college instructors. This study was conducted in accordance with the Declaration of Helsinki revised in 1989.

### Neuromedical and mental disorder assessments

All students had passed their physical examinations during their freshman year; thus, we did not use standard physical examinations. We only employed a self-report questionnaire to access their physical health. No subjects in this study had a serious physical illness during their scanning. To assess the potential mental disorders, two well-trained and experienced graduate students in the school of psychology performed the Structured Clinical Interview for the DSM-IV. The students included in the project did not meet the DSM-IV criteria for psychiatric disorders and did not use drugs that could affect brain function (including antidepressant drugs). None of them developed a psychiatric illness between the different scans.

### Experimental design

For each subject, a series of behavioral data was collected. These data include general demographics for the MRI study (e.g., age and sex), widely used questionnaires that access the personality, mental health and emotion and cognitive tasks (e.g., working memory, go/no-go task and Stroop task). We released part of the behavioral data with the neuroimaging data. Other variables will be considered to be made public in the future or when requested by researchers via e-mail. The comprehensive list of the behavioral data collected is provided at our homepage (http://www.qiujlab.com/resource). The information regarding the behavioral variables previously released is summarized in Table 1.

### Repeated measurements

The behavioral variables that were repeatedly measured (at both time 1 and time 3 or at time 1,2,3) and released include the Beck Depression Inventory (BDI)^13^, and the State-Trait Anxiety Inventory (STAI)^14^.

### Non-repeated measurements

We also released behavioral variables that were only measured at Time1. These variables include the Schutte Self-Report Emotional Intelligence Scale^15^, the Combined Raven’s Matrices test (CRT)^16^.

### Image acquisitions

All of the data (Time 1, Time2 and Time3) were collected at the Southwest University Center for Brain Imaging using a 3.0-T Siemens Trio MRI scanner (Siemens Medical, Erlangen, Germany) in the following order: (1) 3D structural MRI (sMRI); (2) Resting-state fMRI; (3) Task-fMRI (only for a subgroup of subjects) (4) Diffusion-weighted imaging (DWI). To be note, we acquired the task fMRI data in some subjects, but for other research purposes that not related to this longitudinal project. Unlike the IMAGEN study, who scanned the whole sample using the same task paradiagm, we just performed several traditional fMRI studies with relateive small sample size (~40). Therefore, we did not release those task fMRI images within the datasite. We called every subject the day before the experiments to ensure their attendance the subsequent day and to require them to refrain from drinking during the day before the scanning and the scanning day. The questionnaires immediately before the scanning confirmed that the subjects did not drink for at least for one day before the scanning.

#### 3D structural MRI

A magnetization-prepared rapid gradient echo (MPRAGE) sequence was used to acquire high-resolution T1-weighted anatomical images (repetition time=1,900 ms, echo time=2.52 ms, inversion time=900 ms, flip angle=9 degrees, resolution matrix=256×256, slices=176, thickness=1.0 mm, voxel size=1×1×1 mm^3^).

#### Resting-state fMRI

During the resting-state MRI scanning, the subjects were instructed to lie down, close their eyes, and rest without thinking about a specific thing, but refrain from falling asleep. The 8-min scan of 242 contiguous whole-brain resting-state functional images was obtained using gradient-echoplanar imaging (EPI) sequences with the following parameters: slices=32, repetition time (TR)/echo time (TE)=2000/30 ms, flip angle=90, field of view (FOV)=220×220 mm, and thickness/slice gap=3/1 mm, and voxel size=3.4×3.4×3 mm^**3**^.

#### Diffusion weighted imaging (DWI)

The diffusion tensor data for each subject were obtained using a diffusion-weighted, single shot, spin-echo, EPI sequence (TR/TE=11000/98 ms, matrix=128×128, field of view (FOV)=256×256 mm, voxel size=2.0×2.0×2.0 mm^3^, 60 axial slices, 2.0 mm slice thickness, b value 1=0 s/mm^2^, b value 2=1000 s/mm^2^) in 30 directions. In fact, we scanned the subjects three times repeatedly to increase the signal-to-noise (SNR) in the DWI sequence. More specifically, each time, we acquired one b0=0 volume and thirty b0=1000 volumes. Therefore, the final NIFTI files have 93 volumes and the directions information can be found in bval and bvec files.

#### Code availability

We shared the custom code we used in the technical validation analyses on github (https://github.com/weiliu1991/SLIM_Technical-Validation-). Any other code we downloaded from the Internet is also available for everyone by visiting the link we attached.

## Data Records

This dataset is publicly available from the Functional Connectomes Project International Neuroimaging Data-Sharing (Data Citation 1). We removed the facial information of each participant the S-MRI data (FullAnonymize.sh V1.0b; http://www.nitrc.org/frs/shownotes.php?release_id=1902), and the Neuroimaging Informatics Technology Initiative (NIFTI) headers according to FCP/INDI policies. To be note, our data license is the CC-BY-NC (http://www.nitrc.org/include/glossary.php#546). The contents and data structures of these packages are detailed as follows:

### Raw MRI data

The MRI data of all participants are stored in the folder with the subjects’ IDs. The participants’ folder may contain 1 or 2 or 3 subfolders depending on how many scan sessions the participants participated (e.g., if the participant finished all three sessions, his or her folder should include three folders—session 1, session 2, session 3). Each subfolder includes MRI images at corresponding time points (named session 1 or 2 or 3). The raw MRI data includes an sMRI image (named anat_rpi.nii), rfMRI images (named rest_rpi.nii), and diffusion weighted imgaing (DWI) (named dti_rpi.nii). These data were placed in three separate folders (anat_1, rest_1, and dti_1 respectively). Notably, we also placed the b-values and b-vectors in the dti folder with the names dti_rpi.bval and dti_rpi.bvec, respectively.

### Demographic and behavioral data

Each type of behavioral data was placed in a separate folder. The name of the folder was identical to the measurement used. For example, the data for the Beck Depression Inventory (Beck_Depression_Inventory.csv) was placed in a folder named Beck_Depression_Inventory. It is worth noting that the scan date of each participant at different time points was recorded with the demographic information (Time1_data_check.CSV)

### Quality control report

The folder quality-assessment-protocol com package of QA analysis results performed in the present study for the structural and functional images. Specifically, this package includes three folders (named Time1, Time2, and Time 3 respectively). Each folder has three csv files (named SessionX_qap_anatomical_spatial.csv, SessionX_ qap_functional_spatia.csv, and SessionX_qap_functional_temporal.csv, respectively). Those files were generated by the Preprocessed Connectomes Project Quality Assessment Protocol (http://preprocessed-connectomes-project.github.io/quality-assessment-protocol/) and we did not change any part of the pipeline.

## Technical Validation

### General data quality control

All SLMI data were made available to users regardless of data quality because there are no consensus criteria to determine what type of MRI images should be excluded. To quantitatively access the quality of the MRI data, we also calculated a series of quality metrics that have been used widely in the quality control of the MRI images. The calculation was conducted using the Preprocessed Connectomes Project Quality Assessment Protocol. All metrics computed by the protocol may be found together with the data. The definitions of several important metrics are itemized as follows:

- **Spatial Metrics (sMRI, rsfMRI)**:
- **Signal-to-noise ratio (SNR):** The mean values within the brain are divided by the standard deviation of the air values^17^.
- **Contrast to Noise Ratio (CNR)**: Calculated as the mean of the gray matter values minus the mean of the white matter values, divided by the standard **deviation** of the air values^17^.
- **Entropy Focus Criterion (EFC)**: Uses the Shannon entropy of voxel intensities as an indication of ghosting and blurring induced by head motion^18^.
- **Foreground to Background Energy Ratio (FBER)**: Mean energy of image **values** (i.e., mean of squares) within the head relative to outside the head.
- **Artifact Detection (Qi1)**: The proportion of voxels with an **intensity** corrupted by artifacts normalized by the number of voxels in the background^19^.
- **Temporal Metrics (rsfMRI):**
- **Mean Fractional Displacement—Jenkinson**: A measure of subject head motion, which compares the motion between the current and previous volumes. This measure is calculated by summing the absolute value of the displacement changes in the x, y and z directions and the rotational changes about these three axes. The rotational changes are assigned distance values based on the changes across the surface of an 80 mm radius sphere^18,20^.
- **Number of volumes with FD greater than 0.2 mm**: FD>0.2 mm is considered as a threshold for scrubbing^21^.
- **Median Distance Index**: The mean distance (1—Spearman’s rho) between each time-point’s volume and the median volume using AFNI’s 3dTqual command^22^.
- **Standardized DVARS**: The spatial standard deviation of the temporal derivative of the data, normalized by the temporal standard deviation and temporal autocorrelation^21^.
- **Outlier Detection** [outlier]: The mean fraction of the outliers identified in each volume using the 3dToutcount command from AFNI^22^.

### Results of quality metrics of MRI data

**Figure 2:**
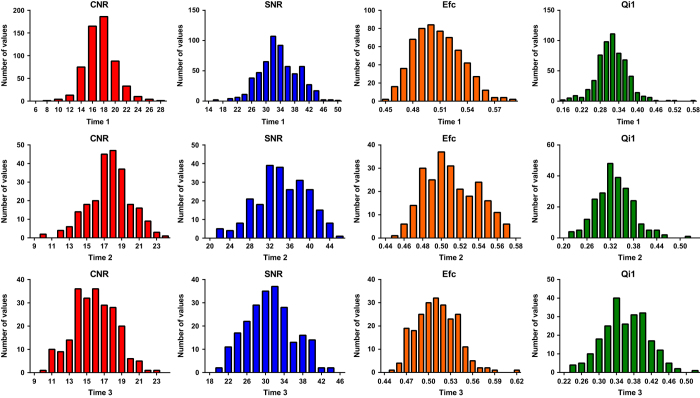
Distributions of quality metrics of structural MRI data.

**Figure 3:**
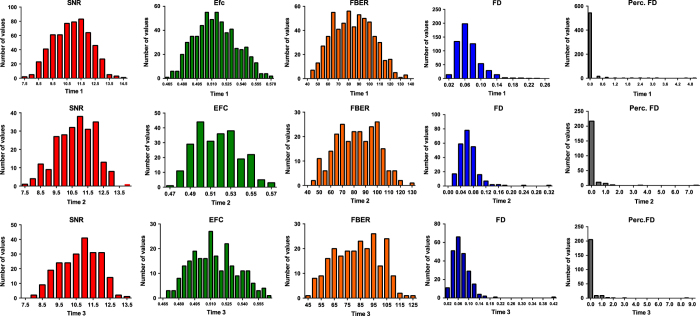
Distributions of quality metrics of resting-state functional MRI data.

Figures 2 and 3 indicate the distributions of the several quality metrics of the structural MRI and resting-state fMRI, respectively, across participants and time points. In addition to these metrics, all metrics that were calculated using the Preprocessed Connectomes Project Quality Assessment Protocol are presented together with the data.

### Relationship between head motion and signal-to-noise ratio (SNR)

To investigate the impact of head motion during the resting-state fMRI scanning on the overall quality of the resting-state images, we correlated the head motion (mean FD) with the signal-to-noise ratio (SNR) in the entire sample (time 1, time 2 and time 3, total *N*=1045). A significant and negative correlation was identified between the mean FD and SNR (r=−0.068, *P*=0.027), and this relationship remained marginally significant (r=−0.058, *P*=0.064) when we removed the subjects with high head motion (mean FD>0.2 mm, *N*=6). Those results suggested that head motion slightly hurts the SNR and the relationship between head motion and image quality appeared to be driven by subjects who have higher head motion (mean FD>0.2 mm).

### Visualization and test-retest reproducibility of functional connectome

**Figure 4:**
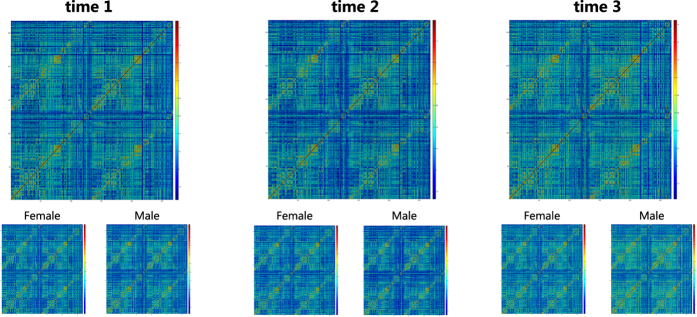
Visualization of functional connectome at three time points for female and male separated and combined.

After common preprocessing steps for resting-state fMRI, we constructed the functional connectome using a widely used functional brain atlas^23^. We calculated the Pearson correlation coefficients between the time courses of each possible pair of nodes and obtained the 268×268 symmetrical connectivity matrices. Each element in the matrices represents a functional connectivity strength between two nodes. As shown in Fig. 4, the patterns of the functional connectome matrices remained stable across different time points. We also calculated the correlation between the matrices at different time points at the subject level (e.g., the correlation between the connectivity matrices at the time 1 and time 2) and subsequently averaged these correlation coefficients at the group level (The code is available at https://github.com/weiliu1991/SLIM_Technical-Validation-). For the subjects who completed three scans (*N*=109), those correlation coefficients were equal to about 0.55 (r_Time1,Time2_=0.54; r_Time1,Time3_=0.56; and r_Time2,Time3_=0.53). The correlation coefficients of subjects who finished at least two scans are similar (r_Time1,Time2_=0.55, *N*=202; r_Time1,Time3_=0.56, *N*=199; and r_Time2,Time3_=0.53, *N*=110)

### Test-retest reproducibility of head motion

**Figure 5:**
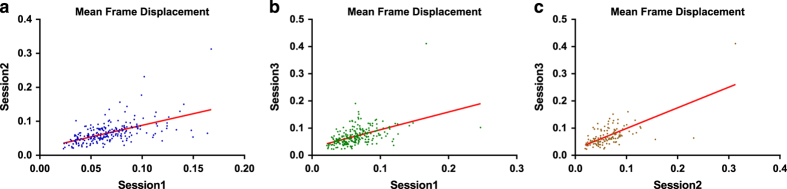
Test-retest plots of head motion during rfMRI scanning. (**a**) The correlation (r=0.562) between the mean FD at Session1 and Session 2. (**b**) The correlation (r=0.492) between the mean FD at Session1 and Session 3. (**c**) The correlation (r=0.671) between the mean FD at Session 2 and Session 3.

Previous studies have demonstrated that head motion has moderate test-retest reliability^7,20,24,25^, which suggests that the head motion may reflect the individual difference. Using the SLIM data, we accessed the long-term test-retest reliability of head motion. The uniqueness of the current analysis is that the interval time between the scans is relatively long (>800 days) and there are subjects who underwent three resting-state fMRI scans within the three and a half years. Similar to previous studies, we found the significant and positive correlation (Fig. 5) between the head motion calculated at different time points (r_Time1,Time2_=0.56, *P*<0.001, *N*=226; r_Time1,Time3_=0.49, *P*<0.001, *N*=227; and r_Time2,Time3_=0.67, *P*<0.001,*N*=123). Significant correlation was identified between the head motion with the long interval (>800 days), thus, the head motion of the subjects may relate to stable individual differences rather than short-term labile variables.

### Relationship between head motion and emotional state

Kong and his colleagues demonstrated that the in-scanner head motion may relate to the a stable psychological trait (impulsivity)^26^. Using the SLIM data, we investigated whether the head motion relates to the subjects’ emotional states (self-reported depression and anxiety symptoms). The mean FD did not correlate with the BDI (r=−0.005, *P*=0.91, *N*=485) or the state anxiety (r=0.062, *P*=0.17, *N*=486) measured immediately after the scanning. These findings further suggested that stable individual differences (e.g., personality) rather than the temporary emotional state contribute to the variability of the in-scanner head motion.

## Usage Notes

Part of this dataset has been successfully used in our previous publications^27–30^. We encourage other labs to use this dataset in publication under the requirement of citing this article and contact us for additional data sharing and cooperation.

All data may be freely downloaded from the International Data-sharing Initiative (INDI) under the CC-BY-NC license. The user of the SLIM should acknowledge the contributions of the original authors and research lab, and properly cite this article.

## Additional Information

**How to cite this article:** Liu, W. *et al.* Longitudinal test-retest neuroimaging data from healthy young adults in southwest China. *Sci. Data* 4:170017 doi: 10.1038/sdata.2017.17 (2017).

**Publisher’s note:** Springer Nature remains neutral with regard to jurisdictional claims in published maps and institutional affiliations.

## Supplementary Material



## Figures and Tables

**Table 1:** Previously Released Behavioral Data.

**Name**	**Type**	**Brief Description**	**Key Variables**	**References**
**Beck Depression Inventory (BDI)**	Repeated	One of the most widely used psychometric tests for measuring the severity of depression	Total score	(Beck *et al.*^13^)
**The State-Trait Anxiety Inventory (STAI)**	Repeated	A commonly used measure of trait and state anxiety	Trait anxiety score; state anxiety score	(Spielberger^14^)
**Schutte Self-Report Emotional Intelligence Scale**	Non-Repeated	a method of measuring general Emotional Intelligence (EI)	Monitoring of Emotions; Utilization of Emotions; Social Ability; Appraisal	(Schutte *et al.*, 1988)
**Combined Raven’s Matrices test (CRT)**	Non-Repeated	A test used to measure abstract reasoning and regarded as a non-verbal estimate of fluid intelligence	Total Score	(Li *et al.*^16^)
